# A novel *NODAL* variant in a young embolic stroke patient with visceral heterotaxy

**DOI:** 10.1186/s12883-024-03619-x

**Published:** 2024-04-11

**Authors:** Kei Kaburagi, Yuta Hagiwara, Keiji Tachikawa, Noriko Miyake, Hisanao Akiyama, Yosuke Kawai, Yosuke Omae, Katsushi Tokunaga, Yoshihisa Yamano, Takahiro Shimizu, Satomi Mitsuhashi

**Affiliations:** 1 Department of Neurology, St. Marianna University School of Medicine, 2-16-1 Sugao, Miyamae-ku, Kawasaki, Kanagawa 2168511 Japan; 2 Department of Human Genetics, Research Institute, National Center for Global Health and Medicine, Tokyo, Japan; 3 Genome Medical Science Project, National Center for Global Health and Medicine, Tokyo, Japan; 4 Department of Rare Diseases Research, Institute of Medical Science, St. Marianna University School of Medicine, Kawasaki, Japan

**Keywords:** Visceral heterotaxy, Cryptogenic stroke, Whole-genome sequencing

## Abstract

**Background:**

Ischemic stroke in young adults can be caused by a variety of etiologies including the monogenic disorders. Visceral heterotaxy is a condition caused by abnormal left–right determinations during embryonic development. We aimed to determine the cause of a young ischemic stroke patient with visceral heterotaxy.

**Case presentation:**

We performed neurological, radiological, and genetic evaluations in a 17-year-old male patient presenting ischemic stroke and visceral heterotaxy to determine the underlying cause of this rare disease combination. Brain magnetic resonance imaging (MRI) showed evidence of embolic stroke, abdominal computed tomography (CT) showed visceral heterotaxy, and echocardiogram showed cardiac anomaly with right-to-left-shunt (RLS). Whole genome sequencing (WGS) revealed a heterozygous missense variant (NM_018055.5: c.1016 T > C, p.(Met339Val)) in the *NODAL* gene, which is essential to the determination of the left–right body axis.

**Conclusions:**

Our study highlights the importance of evaluating genetic etiology in young ischemic stroke and the need for stroke risk management in visceral heterotaxy patients with RLS. To the best of our knowledge, we report the first genetically-confirmed case of visceral heterotaxy with young embolic stroke reported to date.

## Background

Ischemic stroke in young adults is occasionally reported in multiple conditions including monogenic disorders. Genetic testing can find the underlying genetic causes such as defects of coagulation factors, connective tissue abnormalities, or cardiovascular disorders [1, 2]. Despite the impact of monogenic stroke on patients and their families and the importance of assessing genetic causes, patients with young ischemic stroke do not always undergo genetic investigation probably due to their rarity and lack of cost-effective evidence of high-throughput gene sequencing. Efforts have been made to utilize genetic analysis using stroke gene panels [2–4] or whole-exome sequencing [5] to manage monogenic stroke, but the detection rate of the causal genes varies and the clinical significance is unclear probably due to the heterogeneous nature of the stroke and lack of our knowledge when monogenic disorder should be suspected in ischemic stroke.

Young adults with congenital cardiac disease have increased risk of cerebral ischemic stroke. While this is primarily due to electro-abnormalities, such as atrial fibrillation, embolic stroke from the venous system due to the right-to-left shunt may also be a factor even in the absence of arrythmia. Therefore, it may be important to consider the preventive medications in managing the patient. However, there has been no study on cardioembolic stroke due to visceral heterotaxy.

Here we report a patient with visceral heterotaxy and embolic stroke whose genetic testing was useful to identify a novel pathogenic variant in the *NODAL* gene, emphasizing the importance of performing genetic analysis in a patient with young ischemic stroke.

## Case presentation

The patient is a 17-year-old male with asplenia syndrome (complete endocardial deficiency, double outlet right ventricle, and pulmonary artery stenosis) as a congenital heart malformation, although his symptoms are only mild cyanosis, and he is independent in activities of daily living (ADL). His parents were healthy and he was an only child. He was referred to our hospital with a sudden episode of transient left hemiparesis. He suddenly became aware of weakness and sensory disturbance on the left upper and lower limbs while defecating. The symptoms gradually improved within 3 h, but the sensory disturbance remained. There was no marked family history and his parents were healthy.

**Fig. 1:**
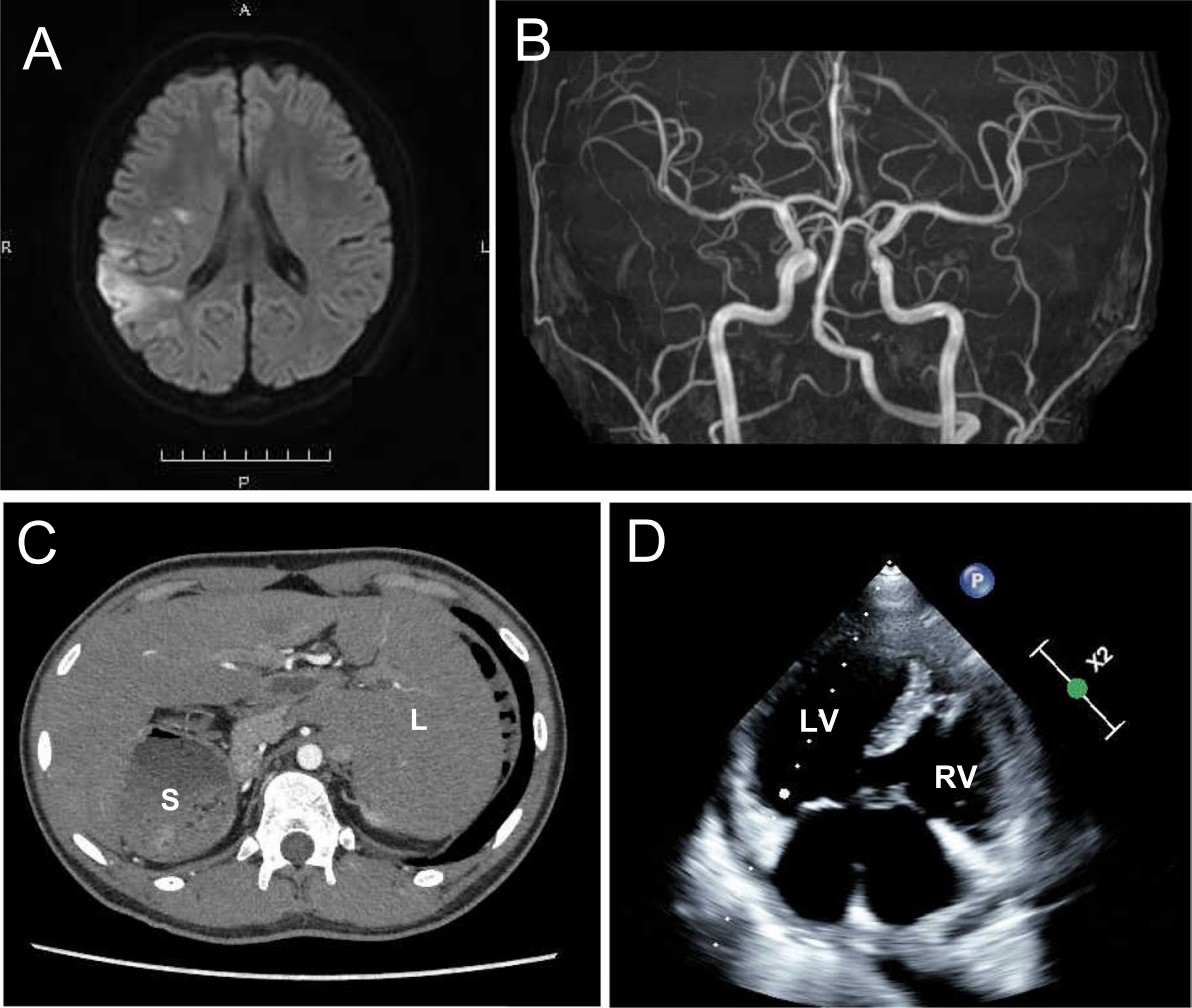
Clinical and radiological features of the patient. **A** The head MRI (Diffusion weighted image) shows acute cerebral infarction on temporo-parietal lobe. **B** MR angiography image shows no vessel abnormality. **C** Abdominal CT shows visceral heterotaxy. S: Stomach. L: Liver. **D** Echocardiogram. Echocardiogram shows ECD. RV: Right ventricle. LV: Left ventricle

We suspected cerebrovascular disease, because he had a cardiac malformation that could cause a right-to-left shunt. The brain MRI showed cerebral infarction on the right temporo-parietal lobe, and magnetic resonance angiography (MRA) showed no vessel abnormalities (Fig. 1A, B). Based on the findings of MRI, we concluded that the patient had an embolic mechanism through cardiac malformation. Abdominal Computer tomography (CT) scan showed visceral heterotaxy (Fig. 1C). Transthoracic echocardiography revealed endocardial cushion defect (ECD) (Fig. 1D). We were not able to perform transesophageal echocardiography. No atrial fibrillation was reported on standard electrocardiogram (ECG) and Holter ECG.

After admission at the age of 17, blood tests showed slightly low activity of antithrombin III (61%) and protein C (63%), and the presence of congenital coagulopathy was suspected, although genetic testing for *SERPINC1* and *PROC* were negative.　In addition, there was no evidence of antiphospholipid antibody syndrome or hyper-homocystinemia, which could cause juvenile cerebral infarction. There were no signs of infectious endocarditis and of deep vein thrombosis (DVT). However, the level of D-dimer FDP was slightly elevated 0.6 μg/ml (normal range: 0–0.5 μg/ml) and the Risk of Paradoxical Embolism (RoPE) score [6] was high (score = 10). Therefore, we diagnosed him as a paradoxical cerebral embolism caused by ECD and potential DVT with right-to-left shunt.

**Fig. 2:**
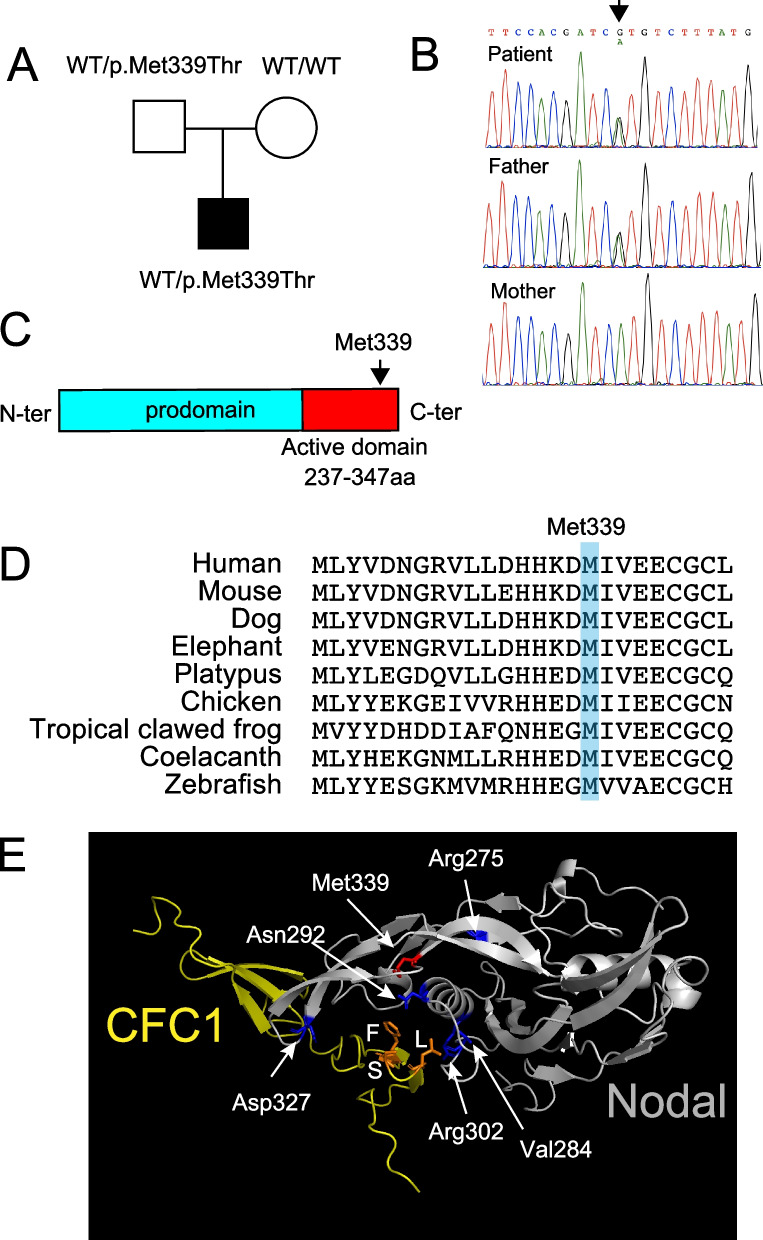
Genetic analysis of *NODAL* variant. **A** Family tree of the patient. WT: wild-type (**B**) Sanger-sequencing validated c.1016 T > C variant in the patient and the father. **C** The scheme of NODAL protein. Nodal is post-translationally cleaved and forms mature active ligand (red). Met339 is located in the active ligand (arrow). **D** Met339 and nearby residues are conserved across species. Genome alignment was obtained from UCSC genome browser (http://genome.ucsc.edu). **E** Evaluation of protein structure of M394 (red) and other residues (blue) on the TGF-β domain, whose changes are reported to cause heterotaxy. Protein structures of homo-dimerized Nodal mature protein (gray) and CFC1-EGF domain of CFC1 (yellow) were modeled by Alphafold2. L, S and F (orange) in CFC1 represent the three residues reported to be essential for binding to Nodal in CFC1 of mouse Cripto [11]. The molecular structure was drawn by PyMOL (www.pymol.org). Amino acids reported to cause visceral heterotaxy including M339 cluster at the CFC1 and Nodal binding site

To further investigate the cause of his stroke, whole-genome sequencing (WGS) was performed on the patient (Fig. 2A, See online method). WGS of the patient revealed a novel heterozygous variant NM_018055.5: c.1016 T > C: p.(Met339Thr) (chr10:70,432,964–70432964 in the genomic coordinate of GRCh38) in the *NODAL* gene (NM_018055.5, MIM#601,265) (Fig. 2B), which is responsible for autosomal dominant visceral heterotaxy (MIM#270,100) [7]. The obtained coverage of WGS was 33.5 x. The unaffected father also had this variant, suggesting incomplete penetrance as reported [7] (Fig. 1A). The father underwent an abdominal CT scan that was completely normal. Sanger-sequencing validated the variant (Fig. 1B). This variant has not been reported in the gnomAD database or the WGS database of 9850 Japanese control individuals [8]. The missense variant was predicted to be damaging with CADD-PHRED score of 26.2. This variant was located in the evolutionally-conserved Nodal active ligand (110 aa) (Fig. 2C, D) and showed high conservation scores (GERP of 5.84, SIFT of 0, PhyloP of 4.603, PolyPhen-2 of 0.998, and PhastCons of 1). MutationTaster prediction was “Disease causing”. Alteration of the same amino acid, c.1015A > G, p.(Met339Val), was reported to cause a mild reduction in nodal signaling by using activin-responsive ARE-luciferase reporter assay in a zebrafish embryo, although detailed clinical description of the patient with the Met339Val variant was unavailable [9]. Protein structure of homodimerized Nodal and its co-receptor CFC1 (CFC1-EGF domain) was modeled by AlphaFold2 [10], which showed that Met339 and other previously reported pathogenic variants were located at the Nodal-CFC1 binding site (Fig. 2E). We further searched for pathogenic variants of the *PROC* gene in the WGS data that may explain the decrease in Protein C in this patient, but were unable to find any candidate variants. Furthermore, we did not identify any pathogenic variants that were responsible for monogenic stroke panel genes [2].

Anticoagulation therapy was started with continuous intravenous unfractionated heparin and switched to warfarin. The patient showed no recurrence of ischemic stroke for one and a half years as of now.

## Discussion and conclusions

We described a novel *NODAL* missense variant in a patient presenting with young embolic stroke and visceral heterotaxy. This variant was located in a highly conserved Nodal active domain, and analysis of the protein structure suggested that it may have a role in CFC1 binding. It is noteworthy that the unaffected father has the same variant. Disruption of the left–right axis determination in embryonic development can result in random left–right selection [12]. In support of this, genetic analyses of heterotaxy family showed approximately 50% penetrance, even in monozygotic twins [13]. A heterotaxy family with a *NODAL* variant was reported, suggesting a ~ 50% penetrance rate [14]. Collectively, it is likely that the unaffected father with Met339Ther did not show heterotaxy due to random determination of the left–right axis. Family-based genetic analysis using high-throughput sequencers usually uses variant filtering based on a hypothesis of 100% penetrance. It may be important to consider this randomness of disease manifestation when performing genetic analysis in a family with visceral heterotaxy.

The patient had an embolic stroke. We considered whether genetic abnormality and cerebral infarction are related. As echocardiography showed an apparent right-to-left shunt, it may be possible that a right-to-left shunt in the heart allowed clots in the venous system to enter the brain circulation and caused an embolic stroke. Although we could not detect DVT, we suspected that the abnormal finding of protein C level inspires the insidious occurrence of DVT. Although the patient showed a mildly reduced Protein C level (63%), no pathogenic variants were found in the *PROC* gene. It is reported that protein C deficiency due to pathogenic genetic variants can decrease Protain C level below 55%. Mild reductions (55–65%) are also seen in healthy individuals [15]. It may be caused by less effective genetic conditions, such as common eQTL variants in regulatory regions [16], although we could not draw any conclusions about this possibility. Thus, we diagnosed the type of cerebral infarction as paradoxical cerebral embolism. We should be aware that patients with a *NODAL* variant may develop a stroke. Although not all young ischemic stroke patients necessarily require genetic testing, patients with possible monogenic diseases observed in the present case should be considered for obtaining the correct diagnosis and providing genetic counseling.

In conclusion, we report a novel *NODAL* variant in a young embolic stroke patient with visceral heterotaxy. It would be important to investigate a monogenic disorder that may be secondarily related to a young embolic stroke. It is also important to carefully evaluate unaffected family members when performing family-based WGS, as heterotaxy can have a penetrance of 50% due to random left–right selection.

## Data Availability

WGS data is private and unavailable.
